# Proteomic Analysis of a Syntrophic Coculture of *Syntrophobacter fumaroxidans* MPOB^T^ and *Geobacter sulfurreducens* PCA^T^

**DOI:** 10.3389/fmicb.2021.708911

**Published:** 2021-11-30

**Authors:** Monir Mollaei, Maria Suarez-Diez, Vicente T. Sedano-Nunez, Sjef Boeren, Alfons J. M. Stams, Caroline M. Plugge

**Affiliations:** ^1^Wetsus, European Centre of Excellence for Sustainable Water Technology, Leeuwarden, Netherlands; ^2^Laboratory of Microbiology, Wageningen University & Research, Wageningen, Netherlands; ^3^Laboratory of Systems and Synthetic Biology, Wageningen University & Research, Wageningen, Netherlands; ^4^Laboratory of Biochemistry, Wageningen University & Research, Wageningen, Netherlands; ^5^Centre of Biological Engineering, University of Minho, Braga, Portugal

**Keywords:** *Syntrophobacter fumaroxidans*, *Geobacter sulfurreducens*, coculture, interspecies electron transfer, propionate, proteomics

## Abstract

We established a syntrophic coculture of *Syntrophobacter fumaroxidans* MPOB^T^ (SF) and *Geobacter sulfurreducens* PCA^T^ (GS) growing on propionate and Fe(III). Neither of the bacteria was capable of growth on propionate and Fe(III) in pure culture. Propionate degradation by SF provides acetate, hydrogen, and/or formate that can be used as electron donors by GS with Fe(III) citrate as electron acceptor. Proteomic analyses of the SF-GS coculture revealed propionate conversion *via* the methylmalonyl-CoA (MMC) pathway by SF. The possibility of interspecies electron transfer (IET) *via* direct (DIET) and/or hydrogen/formate transfer (HFIT) was investigated by comparing the differential abundance of associated proteins in SF-GS coculture against (i) SF coculture with *Methanospirillum hungatei* (SF-MH), which relies on HFIT, (ii) GS pure culture growing on acetate, formate, hydrogen as propionate products, and Fe(III). We noted some evidence for DIET in the SF-GS coculture, i.e., GS in the coculture showed significantly lower abundance of uptake hydrogenase (43-fold) and formate dehydrogenase (45-fold) and significantly higher abundance of proteins related to acetate metabolism (i.e., GltA; 62-fold) compared to GS pure culture. Moreover, SF in the SF-GS coculture showed significantly lower abundance of IET-related formate dehydrogenases, Fdh3 (51-fold) and Fdh5 (29-fold), and the rate of propionate conversion in SF-GS was 8-fold lower than in the SF-MH coculture. In contrast, compared to GS pure culture, we found lower abundance of pilus-associated cytochrome OmcS (2-fold) and piliA (5-fold) in the SF-GS coculture that is suggested to be necessary for DIET. Furthermore, neither visible aggregates formed in the SF-GS coculture, nor the pili-E of SF (suggested as e-pili) were detected. These findings suggest that the IET mechanism is complex in the SF-GS coculture and can be mediated by several mechanisms rather than one discrete pathway. Our study can be further useful in understanding syntrophic propionate degradation in bioelectrochemical and anaerobic digestion systems.

## Introduction

*Geobacter* bacteria are important in soils and sediments containing available organic matter and amorphous Fe(III) (Li et al., 2012; Reguera and Kashefi, 2019). *Geobacter* species play a key role in syntrophic interactions by the removal of fermentation products, such as acetate, formate, and hydrogen, and mitigating the thermodynamic barrier that would otherwise inhibit organic compound decomposition. In these Fe(III)-dependent syntrophic partnerships, *Geobacter* species reduce Fe(III) (hydroxy)oxides by electrons derived from the oxidation of organic matter. Accordingly, several syntrophic interactions involving *Geobacter* species have been documented (Cord-Ruwisch et al., 1998; Summers et al., 2010; Rotaru et al., 2012) and more might be possible where *Geobacter* could drive other microorganisms degrading organic substrates.

Propionate is an important intermediate in anaerobic degradation of organic matter. However, anaerobic oxidization of propionate is thermodynamically more difficult than other intermediates such as butyrate, lactate, and ethanol. Hence, it can accumulate in anaerobic environments such as digesters (Kaspar and Wuhrmann, 1977; Van Lier et al., 1996; Ghasimi et al., 2009; Shah et al., 2009; Liu et al., 2010) and inhibit efficient anaerobic digestion. Therefore, anaerobic conversion of propionate is a prime example of a syntrophic relationship between propionate-oxidizing bacteria and downstream partners, usually methanogens (Worm et al., 2011).

**FIGURE 1:**
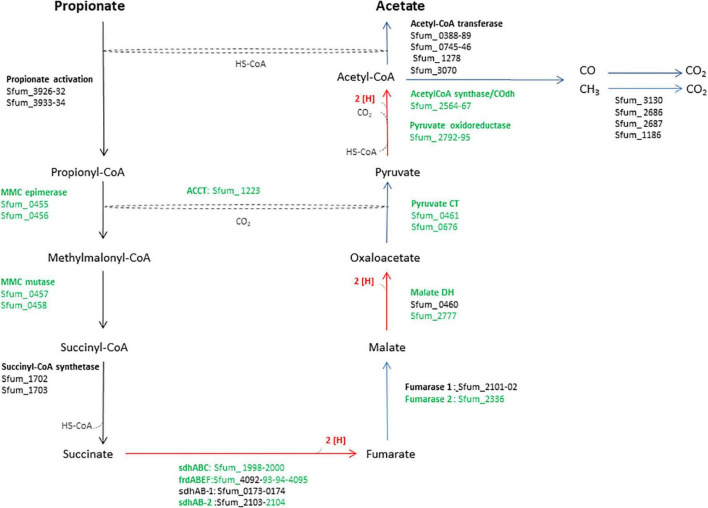
An overview of enzymes and pathway of the methylmalonyl-CoA pathway of SF. The enzymes in green were used in the heatmap in Figure 3. MMC, methylmalonyl-CoA; Sdh, succinate dehydrogenase; Frd, fumarate reductase; DH, dehydrogenase; CT, carboxyltransferase; ACCT, acetyl-CoA carboxyltransferase; CoA Trans, coenzyme A transferase.

*Syntrophobacter fumaroxidans* (SF) is a propionate-oxidizing bacterium that degrades propionate in pure culture using sulfate or fumarate as an electron acceptor (Plugge et al., 1993; Van Kuijk and Stams, 1995; Harmsen et al., 1998), or in syntrophic associations with methanogens such as *Methanospirillum hungatei* (MH) or *Methanobacterium formicicum* (MF) that utilize hydrogen and formate to make propionate oxidation an exergonic process (Harmsen et al., 1998). Therefore, propionate degradation requires hydrogen and formate scavengers such as methanogens to keep these concentrations sufficiently low (1 Pa and 10 μM, respectively) and make the reaction energetically feasible (Dong and Stams, 1995; Schink and Stams, 2006). SF metabolizes propionate using the methylmalonyl-CoA (MMC) pathway (Figure 1) (Plugge et al., 1993). In this pathway, succinate oxidation *via* menaquinone is highly endergonic since the midpoint potential of succinate is much more positive (+30 mV) than the menaquinone (−80 mV) (Plugge et al., 2012). Therefore, this reaction requires a transmembrane proton gradient to function (Plugge et al., 2012). To make the endergonic oxidation of succinate possible, involvement of a periplasmic formate dehydrogenase, cytochrome b:quinone oxidoreductases, the menaquinone loop, and a cytoplasmic fumarate reductase has been proposed (Müller et al., 2010). In the MMC, electrons are produced in three oxidation steps: (i) succinate to fumarate, (ii) malate to oxaloacetate, and (iii) pyruvate to acetyl-CoA plus CO_2_ (red arrows, Figure 1). Consequently, these electrons reduce protons to hydrogen or protons plus CO_2_ to formate. Subsequently, hydrogen and formate are transferred to the methanogenic partner.

Besides methanogens, the syntrophic partner could be any other microbe capable of effective hydrogen and formate removal to facilitate propionate oxidation. *G. sulfurreducens* PCA (GS) can engage in a cooperative partnership with other microbes using different mechanisms for interspecies electron transfer (IET) *via* direct (DIET) and/or hydrogen/formate transfer (HFIT). For instance, HFIT has been identified in cocultures between *Pelobacter carbinolicus* and GS (PC-GS) where ethanol fermentation by *P. carbinolicus* produces hydrogen and formate to support the growth of GS (Rotaru et al., 2012). Furthermore, DIET has been reported in an evolved syntrophic coculture of the ethanol-oxidizing *G. metallireducens* and GS (GM-GS) mediated by extracellular *c*-type cytochromes (Summers et al., 2010). GS can grow using a range of fermentation products such as acetate, formate, and hydrogen. However, fatty acids like propionate and butyrate that are key intermediates in the mineralization of complex organic matter are not used by this bacterium.

A coculture of SF and GS might also overcome the energetic barrier of propionate oxidation. In such a partnership, SF cannot reduce Fe (III) and GS cannot oxidize propionate. Hence, we hypothesized that a coculture benefiting from metabolic interactions between SF and GS might be an alternative strategy for propionate oxidation coupled to Fe(III) by GS. Accordingly, a recent study reported syntrophic propionate degradation by a coculture of SF-GS in the anode of a microbial fuel cell (Wang et al., 2021). Here, we report syntrophic growth of SF-GS cocultures on propionate and Fe(III) followed by proteomic analyses of the coculture to gain insight into the underlying mechanisms of propionate degradation and associated syntrophic interactions.

## Materials and Methods

### Microorganism and Cultivation

*Syntrophobacter fumaroxidans* MPOB (DSM10017) and *G. sulfurreducens* strain PCA (DSM 12127; ATCC 51573) were grown under strict anoxic conditions at 35°C in 120-ml serum bottles with 50 ml of bicarbonate-buffered medium as described previously (Stams et al., 1993). For the pure culture experiments, SF was grown on propionate (20 mM) plus fumarate (60 mM) with N_2_/CO_2_ (80:20, v/v) as the headspace, while GS was grown on a mixture of acetate (5 mM), formate (20 mM), and hydrogen (10 mM) plus Fe(III) citrate (80 mM). Propionate, fumarate, acetate, and formate (all sodium salts) were added from 1 M sterile anoxic stock solutions. To reach 10 mmol hydrogen per liter of liquid medium, 18% of the headspace was filled with pure hydrogen (18.1 kPa). Both bacteria were adapted to their growth conditions by at least five subsequent transfers (10% v/v) to fresh media containing respective electron donors and acceptors.

To construct the coculture, SF and GS were grown on propionate (10 mM) and Fe(III) citrate (80 mM) as electron acceptor in the same medium as the pure cultures. The coculture was adapted to the growth condition by at least five subsequent transfers (10% v/v) in the corresponding media. Growth was determined by analyzing depletion of propionate, production of Fe(II), and increase in volatile suspended solids (VSS) contents. The Fe (III) media were reduced with FeCl_2_ (1.3 mM), and no other reducing agent (e.g., cysteine and sulfide) was used.

### Analytical Methods

Short-chain fatty acids (SCFA) were analyzed using high-performance liquid chromatography (HPLC) using a Dionex UHPLC system (Molenaar et al., 2019). Fe(II) and Fe(III) were quantified with the ferrozine colorimetric method (Stookey, 1970) with absorbance at 562 nm using a U-1500 spectrophotometer (Hitachi, Chiyoda, Tokyo, Japan). The biomass at time zero and at the end of the experiment was analyzed by VSS contents according to standard methods (Clesceri et al., 1995).

### Scanning Electron Microscopy

Biomass samples of the SF-GS coculture were fixed in 2.5% (w/v) glutaraldehyde overnight at 4°C. The fixed samples were washed twice with carbonate/bicarbonate buffer (pH 9, 1.5 M Na^+^) and then dehydrated in a series of ethanol solutions (10, 25, 50, 75, 90, and twice 100%) with 20-min incubations in each step. The slides were dried in a desiccator, coated with gold, and analyzed in a JEOL JSM-6480LV Scanning Electron Microscope. Energy-dispersive X-ray spectroscopy (EDX) analysis was performed using a NORAN Systems SIX (Thermo Scientific, United States).

### Proteomic Analyses

The SF-GS cocultures were used for whole-cell proteomic analyses. Cells from triplicate cultures were harvested at the end of the exponential growth phase by centrifugation at 16,000 *g* for 20 min at 4°C. Cell pellets were washed twice with 20 mM Tris-HCl (pH 7.5) and stored at −80°C until further use. For protein extraction, cell pellets were resuspended in 0.5 ml of SDT-lysis buffer composed of 100 mM Tris/HCl, pH 7.5, 4% w/v sodium dodecyl sulfate (SDS), SIGMAFAST™, Protease Inhibitor Cocktail Tablet (Sigma-Aldrich, Missouri), and 0.1 M dithiothreitol (DTT). Protein extractions, separation, tryptic digestion, and analysis were performed as described previously (Mollaei et al., 2021). The proteins of SF and GS were downloaded from UniProt^1^. An additional database with protein sequences of common contaminants (trypsin, human keratins, and bovine serum albumin) was also included in the database search. False discovery rate (FDR) of less than 1% were set at both peptide and protein levels. The proteomics result contained proteins with at least two identified peptides of which at least one is unique and at least one is unmodified. The natural logarithm was taken from protein label-free quantitation (LFQ; normalized with respect to the total amount of protein and all of its identified peptides) intensities. The non-existing LFQ intensity values were replaced with values obtained by applying a normal distribution down shift of 1.8 and a width of 0.3 (Perseus default values). Relative protein quantification of sample to control was conducted with PERSEUS v.1.6.2.1. by applying two-sample *t* tests using the “log LFQ intensity” columns obtained with an FDR threshold set to 0.05 and S0 = 1. Proteins significantly (*p* < 0.05) present under the given condition were mentioned throughout the text. To determine the proteins’ fold change between two conditions, the log protein abundance ratio of those conditions was considered. Further statistical analyses were performed using R (R Core Team, 2016) and the Venn Diagram package (Chen and Boutros, 2011) as described previously (Mollaei et al., 2021). The proteins SF and GS are listed in the Supplementary Files 1, 2. The mass spectrometry proteomics data have been deposited to the ProteomeXchange Consortium *via* the PRIDE (Vizcaíno et al., 2016) partner repository with the dataset identifier PXD027104.

## Results and Discussion

### Propionate Oxidation and Fe(III) Reduction by the SF-GS Coculture

**FIGURE 2:**
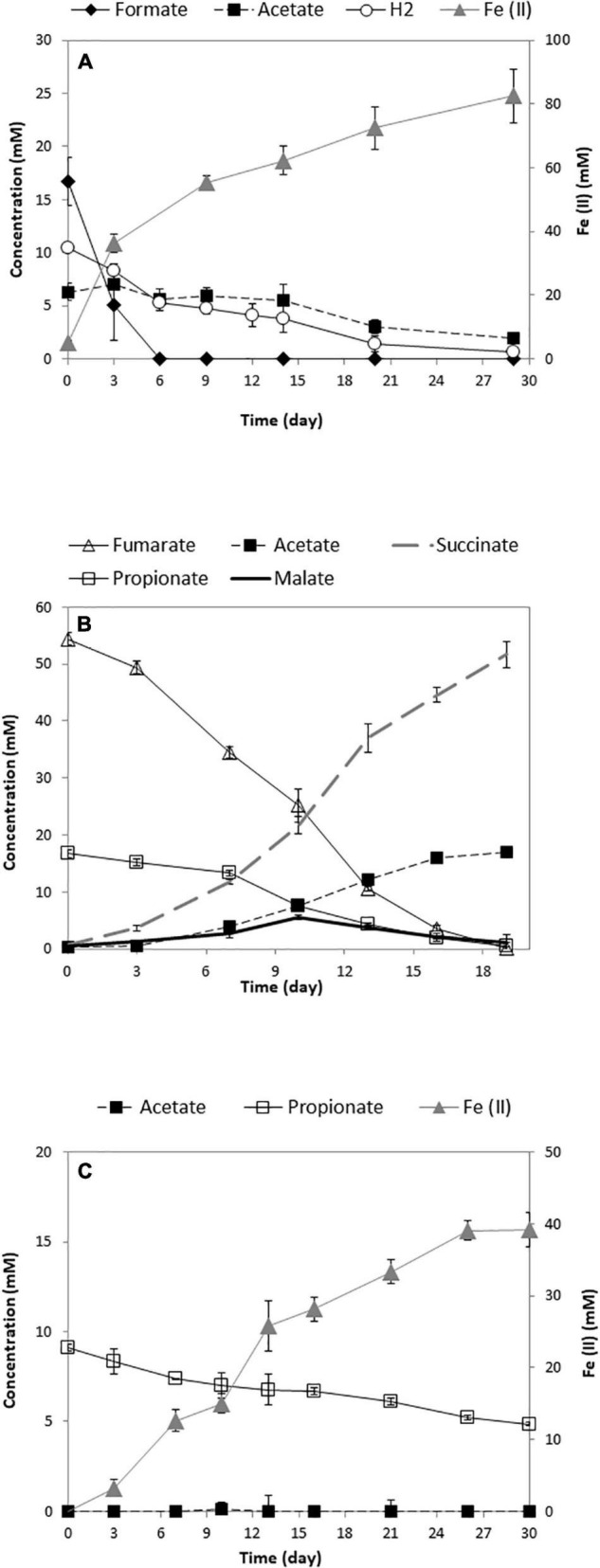
Growth of the pure culture of GS **(A)** (Mollaei et al., 2021), SF **(B)**, and the SF-GS coculture **(C)**. The concentration of hydrogen was shown as mmol/L liquid. Data are means of triplicates.

A syntrophic coculture of the propionate-oxidizing bacterium SF with the Fe(III)-reducing GS was established. Both bacteria are important in anaerobic environments and are known to establish syntrophic interactions with other microbes. Recently, syntrophic propionate degradation using a coculture of SF-GS was reported in a microbial fuel cell (Wang et al., 2021). In our study, we established syntrophic propionate degradation coupled to Fe(III) reduction using a SF-GS coculture. Whereas anaerobic propionate degradation is an endergonic reaction, removal of acetate, formate, and hydrogen by GS coupled to Fe(III) reduction would make the reaction exergonic (Table 1). Our recent physiological and proteomic analyses indeed verified degradation of acetate, formate, and hydrogen by a pure culture of GS (Figure 2A) (Mollaei et al., 2021).

**TABLE 1:** Potential reactions during syntrophic growth of SF-GS coculture based on Gibbs free energy changes.

Reactions	Δ*G*^0^ (kJ/mol)
**Propionate degradation by SF**	
Propionate^–^ + 3H_2_O → acetate^–^ + HCO_3_^–^ + H^+^ + 3H_2_	+76.5
Propionate^–^ + 2HCO_3_^–^ → acetate^–^ + H^+^ + 3HCOO^–^	+72.4
**Acetate, hydrogen, and formate consumption by GS**	
Acetate^–^ + 4H_2_O + 8 Fe^3+^ → 2HCO_3_^–^ + 8Fe^2+^ + 9H^+^	−808.6
H_2_ + 2Fe^3+^ → 2H^+^ + 2 Fe^2+^	−228.3
Formate^–^ + H_2_O + 2Fe^3+^ → HCO_3_^–^ + 2Fe^2+^ + 2H^+^	−226.9
**Syntrophic propionate degradation with removal of acetate (a), hydrogen (b), or formate (c)**	
(a) Propionate^–^ + 7H_2_O + 8Fe^3+^ → 3HCO_3_^–^ + 10H^+^ + 3H_2_ + 8Fe^2+^	−733.3
(b) Propionate^–^ + 3H_2_O + 6Fe^3+^ → acetate^–^ + HCO_3_^–^ + 7H^+^ + 6Fe^2+^	−609.4
(c) Propionate^–^ + 3H_2_O + 6Fe^3+^ → acetate^–^ + HCO_3_^–^ + 7H^+^ + 6Fe^2+^	−609.2
**Complete propionate degradation**	
Propionate^–^ + 7H_2_O + 14Fe^3+^ → 3HCO_3_^–^ + 16 H^+^ + 14Fe^2+^	−733.3

*Data were obtained from Thauer et al. (1977) and Müller et al. (2010).*

Propionate consumption and Fe(III) reduction indicated a successful syntrophic relationship between these two partners (Figure 2C). However, the rate of propionate consumption by the SF-GS coculture (0.14 mM/day) (Figure 2C) was considerably lower than the SF pure culture growing on propionate and fumarate (1.05 mM/day) (Figure 2B) and a SF-MH coculture growing on propionate (1.08 mM/day) (Dong et al., 1994; Sedano-Núñez et al., 2018). Accordingly, propionate oxidation by the SF-GS was 7.5 times lower than the SF pure culture and 8 times lower than the SF-MH coculture. Acetate did not accumulate in the SF-GS coculture (Figure 2C), likely due to acetate consumption by GS. Formate was not detected, while hydrogen was not measured. Propionate degradation also did not continue during extended incubation (data not shown). This may be caused by the inhibitory effect of Fe compounds on methanogenesis in general (Baek et al., 2019). However, this needs to be investigated for SF. Moreover, SF or GS did not grow in pure culture provided with propionate plus Fe(III) citrate.

The Fe(II) produced (39.1 ± 2.4 mmol; mean ± SD, *n* = 3) from 4.3 ± 0.2 mmol propionate consumed (Figure 2C) corresponded to a molar yield of Fe(II) production on propionate of 9.2 ± 0.9. This is consistent with the stoichiometry for the oxidation of propionate to acetate by SF and Fe (III) reduction of acetate by GS (Table 1). Acetate production from propionate by a SF-GS coculture in a microbial fuel cell was also shown by Wang et al. (2021). The biomass was determined by measuring VSS contents of the coculture (3.3 ± 0.6 mg of VSS/mmol propionate consumed). Considering a protein content of 46% of cell dry weight (Mahadevan et al., 2006), the increase in total protein content (129 ± 24 mg/L) indicates growth of the SF-GS coculture in the presence of Fe(III) citrate. In a recent study, growth of a SF-GS coculture in a microbial fuel cell was shown by qPCR (Wang et al., 2021). No visible aggregates formed in the SF-GS coculture, but densely packed cells were noted by scanning electron micrographs (SEMs) (Supplementary Figure 1). This physical contact between electron-donating and electron-accepting partners is important in DIET and for HFIT to make shorter diffusion distances for hydrogen and formate transfer.

### Proteomic Analyses of the SF-GS Coculture

To gain further insights into the metabolism of the SF-GS coculture and to evaluate electron transfer mechanisms, we performed proteomic analyses. Three biological replicates of cocultures were prepared in batch culture. These replicates were harvested when ∼50% of Fe(III)-citrate (80 mM) had been reduced to Fe(II). The proteome of the SF-GS coculture was compared with the previously published proteome of (i) the SF pure culture growing on propionate and fumarate (Sedano-Núñez et al., 2018), (ii) the coculture of SF with MH growing on propionate (Sedano-Núñez et al., 2018), and (iii) the GS pure culture growing on products of propionate oxidation (i.e., acetate, formate, and hydrogen) and Fe(III) (Mollaei et al., 2021). To reduce experimental artifacts, all the studies mentioned (Sedano-Núñez et al., 2018; Mollaei et al., 2021) and this study have been performed at the same time, following exactly the same experimental procedures.

The genome of *S. fumaroxidans* contains 4,098 protein coding genes (PCGs) (Plugge et al., 2012) and *G. sulfurreducens* PCA contains 3,430 PCGs (Butler et al., 2012). From the proteome of the SF-GS coculture, a total of 1,802 proteins were detected with two or more peptides, of which at least one peptide is unique (Supplementary Files 1, 2). Principal component analysis (PCA) showed that the protein abundance patterns were reproducible among the triplicates of the SF-GS coculture (Supplementary Figure 3). The relative abundance of detected proteins from SF and GS revealed that GS proteins accounted for a higher percentage (81.9%) in the cocultures (Supplementary Files 1, 2).

### Proteome of SF in SF-GS Coculture vs. SF Pure Culture and SF-MH Coculture

#### Enzymes of the Methylmalonyl CoA Pathway

**FIGURE 3:**
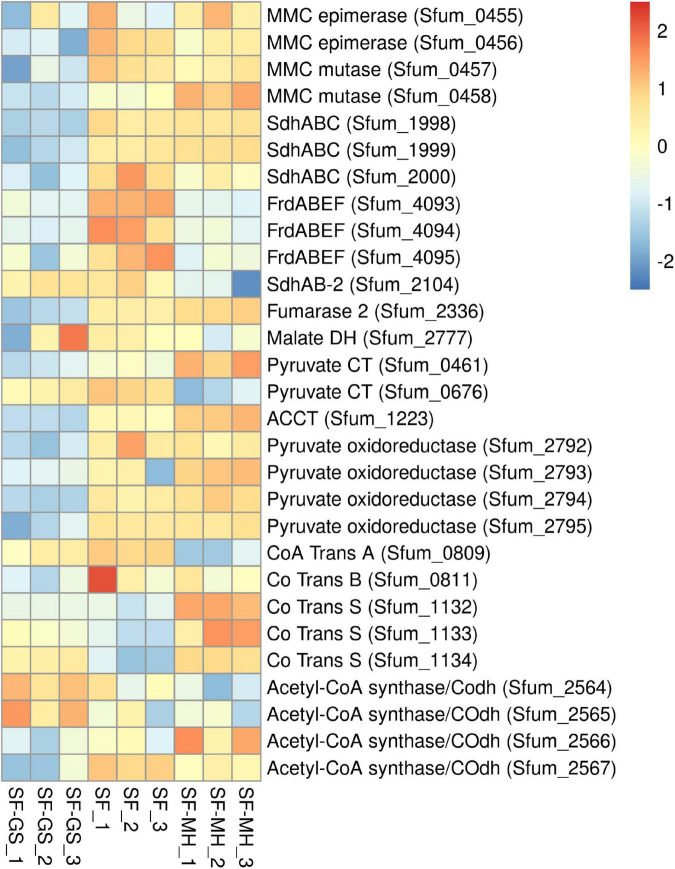
Relative abundance of the detected proteins in the methylmalonyl-CoA pathway of SF in the SF-GS coculture versus the SF pure culture and the SF-MH coculture. Protein abundance levels are shown after *Z*-score normalization. The colour intensity indicates the degree of protein presence, where high relative abundance is indicated in red and low relative abundance in blue. The rows in the heat map show the detected proteins in the SF-GS coculture, the SF pure culture and the SF-MH coculture. The columns show the cultures in triplicates. Clustering shows that samples grouped according to treatments even when only enzymes in this set are considered (Supplementary Figure 6).

Previous genomic analyses of SF predicted several genes coding for proteins involved in the MMC pathway (Müller et al., 2010; Plugge et al., 2012) (Figure 1). Most of these proteins were found in the whole proteome of the SF-GS coculture, albeit with lower abundance as opposed to the pure culture of SF or the SF-MH coculture reported previously (Sedano-Núñez et al., 2018) (Figure 3). Some predicted proteins were not detected in the present study (proteins shown in black in Figure 1) such as the predicted enzymes for propionate activation (Sfum_3926–3934) and for the conversion of acetyl-CoA to acetate by Acetyl-CoA hydrolase (Sfum_0388–0389, Sfum_0745–0746, Sfum_1278, and Sfum_3070). This is consistent with the findings from the SF pure culture and the cocultures of SF with MH and MF (Sedano-Núñez et al., 2018). However, we found paralogous proteins that are likely involved in propionate activation and/or acetate formation in the MMC pathway, i.e., three CoA transferases [CoA-A (Sfum_0809–0810), CoA-B (Sfum_0811–0812), and CoA-S (Sfum_1132–1134)] (Sedano-Núñez et al., 2018) (Figure 3). The abundance of the detected subunits in the SF-GS cocultures are CoA-A (Sfum_0809; 8-fold vs. SF-MH and 3-fold lower vs. SF), CoA-B (Sfum_0811; 5-fold lower vs. SF-MH, 9-fold lower vs. SF), and CoA-S (226-, 32-, and 100-fold significantly lower vs. SF-MH, respectively) (1-, 3-, and 3-fold higher vs. SF, respectively) (Figure 3).

Succinate oxidation to fumarate by a membrane-bound succinate dehydrogenase/fumarate reductase and *via* a menaquinone is the most energy-dependent reaction in the MMC pathway (Plugge et al., 1993). The genome of SF contains four gene clusters [sdhABC (Sfum_1998–2000), frdABEF (Sfum_4092–4095), sdhAB-1 (Sfum_0172–0174), and sdhAB-2 (Sfum_2103–2104)] similar to the defined succinate dehydrogenases/fumarate reductase of SF (Plugge et al., 2012). SF might use separate enzymes for succinate oxidation and fumarate reduction (Plugge et al., 2012). SF needs an active fumarate reductase during growth with propionate and fumarate, whereas an active succinate dehydrogenase is required during growth with propionate and sulfate, or during syntrophic growth with propionate (Plugge et al., 1993). Previous genomic analyses (Müller et al., 2010; Plugge et al., 2012) predicted membrane-bound succinate dehydrogenase SdhABC (Sfum_1998–2000) as the main protein complex responsible for the oxidation of succinate to fumarate. However, this enzyme was shown to be active in both directions, i.e., fumarate reduction and succinate oxidation (Van Kuijk et al., 1998). The three subunits of the SdhABC complex showed significantly lower abundance in the SF-GS coculture compared to the other conditions (12-, 8-, and 5-fold vs. SF-MH, respectively) (13-, 7-, and 32-fold vs. SF, respectively).

During SF growth on propionate with fumarate, propionate is converted to succinate (black arrows, Figure 1), and then, fumarate is partially oxidized to acetate (blue arrows, Figure 1). This conversion is energy demanding and produces reducing equivalents during malate oxidation, and pyruvate decarboxylation, and is only possible by its coupling to the energy-yielding reduction of fumarate to succinate. In line with this, three subunits of the fumarate reductase complex, FrdABEF (Sfum_4093–95), were detected in significantly higher abundance (11-, 45-, and 15-fold, respectively) in the SF pure culture versus the SF-GS coculture (Figure 3). Yet, the FrdABEF complex showed slightly higher abundance in the SF-GS coculture compared to the SF-MH coculture (2-, 2-, and 1-fold, respectively) (Figure 3). In line with the former, transcription analyses of the SF pure culture grown with fumarate also reported upregulation of FrdABEF (>2 log ratio) and downregulation in SF cells cocultured with MH (Worm, 2010). Succinate was not seen accumulated in the SF-GS coculture (Figure 2C) similar to SF-MH cocultures (Stams et al., 1993; Sedano-Núñez et al., 2018). In the genome of SF, two additional gene clusters show similarity to succinate dehydrogenases: SdhAB-1 (Sfum_0172–0174) and SdhAB-2 (Sfum_2103–2104). SdhAB-1 was not detected in this study, which is consistent with results from the SF coculture with methanogens (Sedano-Núñez et al., 2018). However, the alpha subunit of SdhAB-2 (Sfum_2104) was detected in this study that was slightly more abundant in the SF-GS coculture (1-fold vs. SF, 10-fold vs. SF-MH) (Figure 3).

Fumarase converts fumarate to malate and maintains the level of fumarate low to pull the oxidation of succinate to fumarate. The predicted fumarase in the gene cluster (Sfum_2101–2102) was not detected in the coculture of SF-GS. Instead, a second fumarase from a non-clustered gene (Sfum_2336) was detected in our study with significantly lower abundance compared to SF-MH coculture (10-fold) and SF pure culture (4-fold) (Figure 3).

The remaining enzymes of the MMC pathway such as methylmalonyl-CoA epimerase (Sfum_0455–0456), methylmalonyl-CoA mutase (Sfum_0457–0458), and pyruvate carboxyltransferase (Sfum_0461 and Sfum_0676) showed lower abundance in the SF-GS coculture compared to the SF-MH coculture and the SF culture (Figure 3). Two enzymes showed significantly lower abundance in the SF-GS coculture, i.e., pyruvate ferredoxin oxidoreductase (Sfum_2792–95) and carboxyl transferase (Sfum_1223) (Figure 3). In contrast, two enzymes showed slightly higher abundance in the SF-GS compared to SF-MH and SF, i.e., malate dehydrogenase (Sfum_2777) (8-fold higher vs. SF-MH, and 1-fold higher vs. SF) and two subunits of acetyl-CoA synthase/COdh complex (Sfum_2564-65) (19- and 15-fold higher vs. SF-MH, and 3- and 7-fold higher vs. SF, respectively) (Figure 3).

#### Potential Involvement of Hydrogenases and Formate Dehydrogenases, Pilin, and Cytochromes of SF in Interspecies Electron Transfer

**FIGURE 4:**
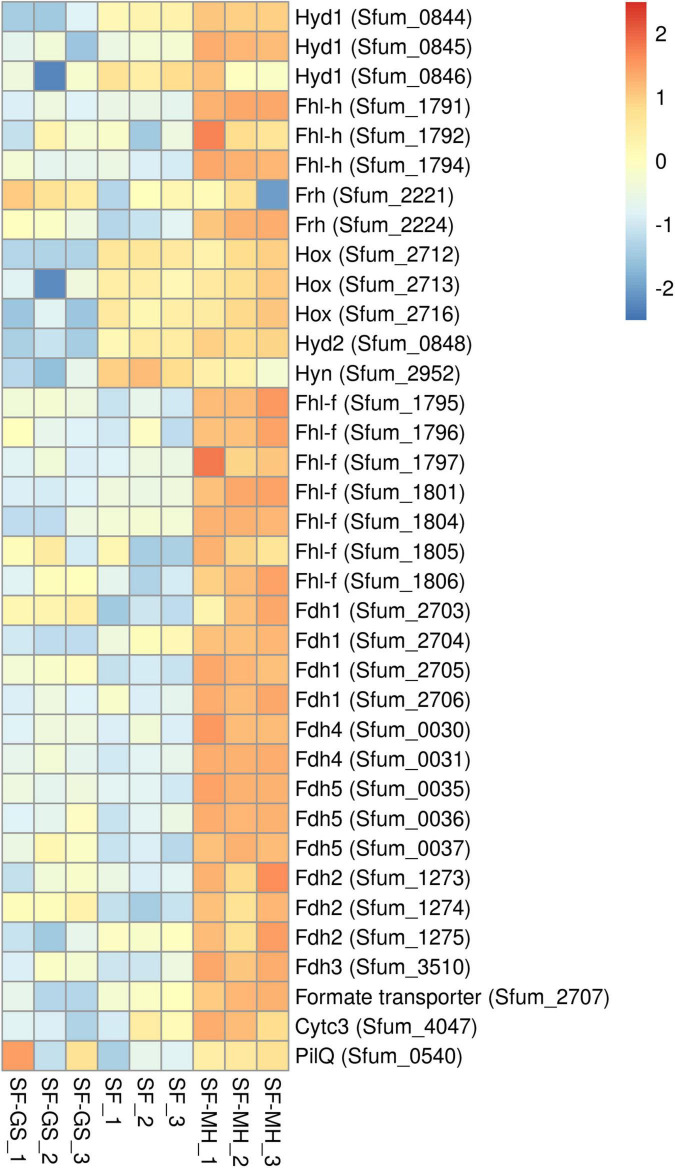
Relative abundance levels of detected hydrogenases and formate dehydrogenases of SF in the SF-GS coculture versus the SF pure culture and the SF-MH coluture. Protein abundance levels are shown after *Z*-score normalization. Protein abundance levels are shown after *Z*-score normalization. The colour intensity indicates the degree of protein presence, where high relative abundance is indicated in red and low relative abundance in blue. The rows in the heat map show the detected proteins in the SF-GS coculture, the SF pure culture and the SF-MH coulture. The columns show the cultures in triplicates. Clustering shows that samples grouped according to treatments even when only enzymes in this set are considered (Supplementary Figure 6).

The genome of SF contains eight hydrogenases and six formate dehydrogenases (Supplementary File 2 and Supplementary Table 6). Of the eight predicted hydrogenases, seven were detected in the present study (Figure 4). The three important cytoplasmic hydrogenases, i.e., Hyd1 (Sfum_0844–0846), Hox (Sfum_2712–2716), and Fhl-h (Sfum_1791–94) detected in this study showed significantly lower abundance in the SF-GS coculture (Figure 4). Other cytoplasmic hydrogenases, Mvh1 (Sfum_3535–3537), Mvh2 (Sfum_3954–3957), and Frh (Sfum_2221–2224), were not found in our study except one subunits of Frh (Sfum_2221) (Figure 4). Sfum_2221 subunit in SF-GS showed slightly higher abundance (2 vs. SF and 3-fold vs. SF-MH) (Figure 4).

The two predicted periplasmic hydrogenases [Hyd2 (Sfum_0847–0848) and Hyn (Sfum_2952–2953)] detected in this study showed significantly lower abundance in the SF-GS coculture compared to the other conditions (Figure 4). Hyd1 might be involved in energy conservation as a confurcating hydrogenase, and Hyn has been suggested to be involved in reverse electron transport (RET) coupled with FrdABEF for fumarate reduction or with SdhABC for succinate oxidation (Worm et al., 2011; Sedano-Núñez et al., 2018). Sedano-Núñez et al. (2018) suggested that hydrogen *via* Hyd1, Hyd2, Hox, and Hyn plays an important role in energy conservation by RET. In line with this, the significantly lower abundance of these hydrogenases in the SF-GS coculture compared to the SF pure culture indicates that they are not required for IET. The lower levels of these hydrogenase may indicate that GS is a better hydrogen-scavenger than methanogens. However, it should be noted that the propionate oxidation rate was much lower in the SF-GS coculture than in the SF-MH cocultures and the SF pure culture.

Among the cytoplasmic formate dehydrogenases, Fdh1 (Sfum_2703–2706) and Fdh4 (Sfum_0030–0031) were detected in significantly lower abundance in the SF-GS coculture versus SF-MH. Compared to the SF culture, Fdh1 (Sfum_2703) (7-fold) and Fdh4 (Sfum_0030–0031) (2- and 1-fold, respectively) were more abundant in the SF-GS cocultures whereas the rest of subunits were lower (Figure 4). The three periplasmic formate dehydrogenases of SF, Fdh2 (Sfum_1273–1275), Fdh3 (Sfum_3510-11), and Fdh5 (Sfum_0035-37), detected in our study showed significantly lower abundance in the SF-GS cocultures than SF-MH (Figure 4). However, compared to SF, Fdh2 (Sfum_1274; 8-fold), Fdh3 (Sfum_3510; 2-fold), and Fdh5 (4-, 2-, and 7-fold, respectively) showed higher abundance in the SF-GS coculture. It was proposed that Fdh3 and Fdh5 are specialized in transferring formate to the methanogenic partner, whereas Fdh2 is used for energy conservation as part of the reverse electron transport mechanism associated with succinate oxidation, possibly coupled to SdhABC or FrdABEF (Sedano-Núñez et al., 2018). The significantly lower abundance of Fdh3 and Fdh5 indicates that formate was not likely the main carrier in the SF-GS coculture compared to the SF-MH coculture.

From the membrane-bound Fhl-f (Sfum_1795–1806), seven subunits (1795–1797, 1801, and 1804–1806) were found with significantly lower abundance in the SF-GS compared to the SF-MH coculture. Compared to the SF pure culture, Fhl-f (Sfum_1795–1796 and 1805–1806) showed (7-, 2-, 3-, and 9-fold, respectively) higher abundance in the SF-GS coculture (Figure 4). A possible role for Fhl-f was suggested in hydrogen-formate interconversion during syntrophic growth (Sedano-Núñez et al., 2018). The formate transporter (Sfum_2707) showed significantly lower abundance in the SF-GS coculture (60-fold vs. SF-MH, 16-fold vs. SF) (Figure 4). This also reflects the low possibility of formate transformation and FIT in the SF-GS coculture. Overall, hydrogenases and formate dehydrogenases were less abundant in the SF-GS coculture compared to the SF-MH coculture (Figure 4). Energetically, Fe(III) reduction creates lower hydrogen and formate levels than methanogenesis; hence, lower enzyme levels are needed to obtain the same rate. However, the lack of methods made it difficult to find the extent of interspecies hydrogen/formate transfer (Rotaru et al., 2014b).

SF has not been observed to grow syntrophically by DIET. In order to provide insight into the potential role of pili and c-type cytochromes of SF in DIET (Supplementary File 2 and Supplementary Table 2), the differential abundance levels of detected ones in SF-GS cocultures were compared against SF-MH that relies on HFIT. PilE (suggested as e-pili) was not detected. PilQ (Sfum_0540) in SF-GS coculture showed slightly lower abundance (1-fold) compared to the SF-MH coculture, but higher abundance (5-fold) compared to the SF pure culture. This may not necessarily indicate a role of PilQ in IET. PilQ might also be produced for adhesion of the cells. *Cytc*_3_ (Sfum_4047) showed lower abundance in the SF-GS coculture (25-fold vs. SF-MH, 8-fold vs. SF) which indicates that its function is not related to electron transfer between SF and GS. Further physiological and gene knockout studies are required to determine if cytochromes/pilin directly contribute to DIET. For instance, PilE/PilQ-deficient mutant of SF cocultures with GS could reveal whether it is associated with DIET.

### Proteome of GS in the SF-GS Coculture vs. GS Pure Culture Grown on Products of Propionate Conversion

The growth of GS pure culture on products of propionate oxidation (acetate, formate, and hydrogen) revealed that when all three substrates are available, formate and hydrogen were the preferred substrates than acetate (Figure 2A, till day 6) (Mollaei et al., 2021).

#### Proteins Related to Acetate Metabolism in GS

**FIGURE 5:**
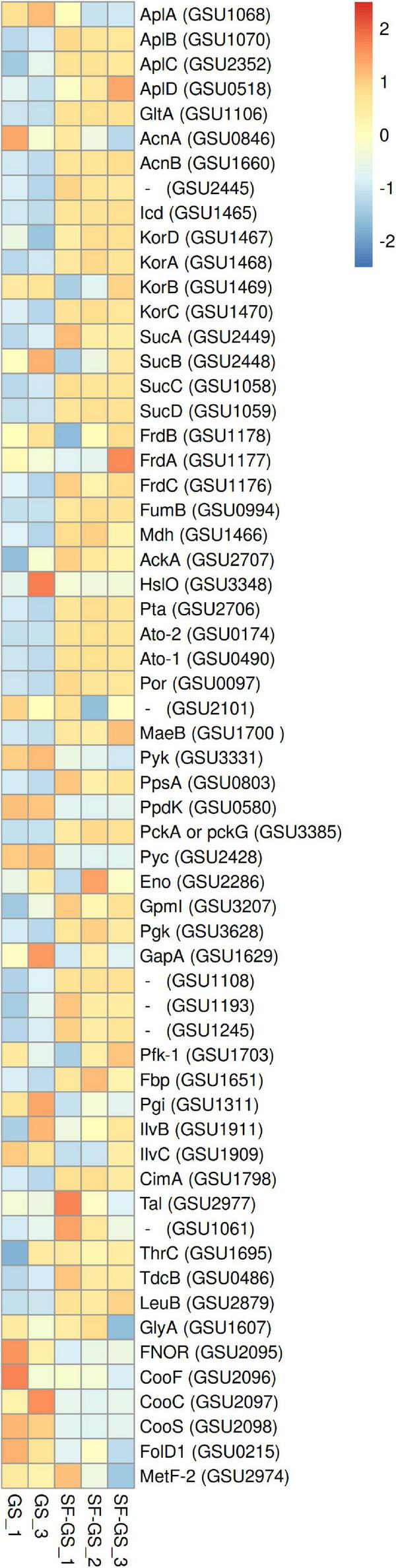
Relative abundance of the detected proteins in the central metabolic network of GS in the SF-GS coculture versus the GS pure culture. Protein abundance levels are shown after *Z*-score normalization. The color intensity indicates the degree of protein presence, where high relative abundance is indicated in red and low relative abundance is indicated in blue. The rows in the heat map show the detected proteins in the SF-GS coculture and the GS pure culture. The columns show the cultures in replicates. The abbreviations of the proteins are as follows: sodium/solute symporter family protein (AplA, AplB, AplC, and AplD), citrate synthase (GltA), aconitate hydratase 1 (AcnA), aconitate hydratase 2 (AcnB), aconitate hydratase, putative (GSU2445), isocitrate dehydrogenase, NADP-dependent (Icd), 2-oxoglutarate:ferredoxin oxidoreductase, ferredoxin subunit (KorD), 2-oxoglutarate:ferredoxin oxidoreductase, alpha subunit (KorA), 2-oxoglutarate:ferredoxin oxidoreductase, thiamin diphosphate-binding subunit (KorB), 2-oxoglutarate:ferredoxin oxidoreductase, gamma subunit (KorC), 2-oxoglutarate dehydrogenase, E1 protein (SucA), 2-oxoglutarate dehydrogenase, E2 protein, dihydrolipoamide succinyltransferase (SucB), succinyl-CoA synthetase, beta subunit (SucC), succinyl-CoA synthetase, alpha subunit (SucD), succinate dehydrogenase/fumarate reductase, iron-sulfur protein (FrdB), succinate dehydrogenase/fumarate reductase, flavoprotein subunit (FrdA), succinate dehydrogenase/fumarate reductase, cytochrome b558 subunit (FrdC), fumarate hydratase, class I (FumB), malate dehydrogenase, NAD-dependent (Mdh), acetate kinase (AckA), chaperonin Hsp33 (HslO), phosphate acetyltransferase (Pta), succinyl:acetate coenzyme A transferase (Ato-2), succinyl:acetate coenzyme A transferase (Ato-1), pyruvate:ferredoxin/flavodoxin oxidoreductase (Por), pyruvate dehydrogenase E1 component subunit alpha (PdhA), pyruvate dehydrogenase E1 component subunit beta (PdhB), formate acetyltransferase/glycerol dehydratase, putative (GSU2101), NADP-dependent malic enzyme (MaeB), pyruvate kinase (Pyk), phosphoenolpyruvate synthase (PpsA), pyruvate phosphate dikinase (PpdK), phosphoenolpyruvate carboxykinase, GTP-dependent (PckA), pyruvate carboxylase (Pyc), enolase (Eno), phosphoglycerate mutase family protein (GSU1818), phosphoglycerate mutase, 2,3-bisphosphoglycerate-independent (GpmI), phosphoglycerate kinase (Pgk), glyceraldehyde-3-phosphate dehydrogenase, type I (GapA), aldehyde dehydrogenase (GSU1108), ketose-1,6-bisphosphate aldolase, class II, putative (GSU1193), ketose-1,6-bisphosphate aldolase, class II, putative (GSU1245), 6-phosphofructokinase (Pfk-1), 6-phosphofructokinase, ATP-dependent (Pfk-2), fructose-1,6-bisphosphatase (Fbp), glucose-6-phosphate isomerase (Pgi), acetolactate synthase (IlvB), ketol-acid reductoisomerase (IlvC), 2-isopropylmalate synthase (CimA), triose-phosphate isomerase (GSU1628), transketolase, C-terminal subunit (GSU2918), transketolase, N-terminal subunit (GSU2919), transaldolase (Tal), aspartate transaminase (GSU1061), threonine synthase (ThrC), (TdcB), 3-isopropylmalate dehydrogenase (LeuB), (GlyA), NADH oxidase, putative or FAD-dependent pyridine nucleotide-disulfide oxidoreductase family protein (FNOR), carbon monoxide dehydrogenase-associated iron-sulfur cluster-binding oxidoreductase (CooF), carbon monoxide dehydrogenase accessory protein (CooC), carbon monoxide dehydrogenase, catalytic subunit (CooS), sensory box protein (RcoM), bifunctional protein FolD 1 (FolD1), and 5-methyltetrahydrofolate-homocysteine S-methyltransferase and 5,10-methylenetetrahydrofolate reductase (MetF-2). Clustering shows that samples grouped according to treatments even when only enzymes in this set are considered (Supplementary Figure 6).

In SF-GS cocultures, GS has the option to use acetate derived from propionate conversion. Our proteomic analyses of acetate uptake, acetate activation, and citric acid cycle (CAC) proteins indicated that GS was actively metabolizing acetate in the SF-GS coculture. Among the acetate uptake proteins of GS (AplA; GSU1068, AplB; GSU1070, AplC; GSU2352), AplB and AplC were more abundant in the SF-GS coculture (11- and 3-fold, respectively), whereas AplA was more abundant in the GS pure culture (5-fold) (Figure 5). Presence of at least two of these proteins is necessary for acetate uptake (Risso et al., 2008; Mahadevan et al., 2011). Acetate is activated before further oxidation and gluconeogenesis *via* acetate activating proteins, i.e., succinyl-CoA:acetate-CoA-transferase (Ato-1; GSU0490 and Ato-2; GSU0174), acetate kinase (AckA; GSU2707), and phosphotransacetylase (Pta; GSU2706) (Segura et al., 2008). All of these proteins showed higher abundance (13-, 59-, 12-, and 29-fold, respectively) in the SF-GS coculture than the GS pure culture (Figure 5).

In the oxidation route of acetate, the abundance of CAC enzymes (Galushko and Schink, 2000) was significantly higher in the SF-GS coculture than the GS pure culture. For example, citrate synthase (GltA; GSU1106), which is required for entry of acetyl-CoA in the CAC and directly correlated with acetate oxidation in the CAC, was 62-fold higher in the SF-GS coculture compared to the GS pure culture (Figure 5). Recently, we found that GS grown on acetate produced almost 15-fold more citrate synthase than when grown on a mix of acetate, hydrogen, and formate (Mollaei et al., 2021). This may indicate that the level of hydrogen and formate was too low in the SF-GS coculture. The nuo-1 cluster (GSU0338 to GSU0351) encoding one of the two NADH dehydrogenase complexes was highly abundant (≥7-fold) in the SF-GS coculture (Supplementary Figure 4), which is consistent with efficient electron transfer from NADH (produced from CAC) to menaquinone, which in turn delivers electrons to the terminal electron acceptor, Fe(III). Thus, the observed protein patterns suggest that GS in the coculture with SF was actively oxidizing acetate. Accordingly, acetate did not accumulate in the SF-GS coculture (Figure 2C). The availability of hydrogen in the GS pure culture could potentially repress the expression of the genes for acetate metabolism through the regulator HgtR (GSU3364) (Ueki and Lovley, 2010). Consistent with this, gene expression patterns showed the reduced expression of acetate metabolism genes in GS in PC-GS coculture compared to GM-GS coculture most likely due to the presence of hydrogen in PC-GS (Shrestha et al., 2013). In the SF-GS coculture, the higher acetate consumption may hint to the low presence of hydrogen, although hydrogen was not measured in our study. Recently, acetate production and consumption were confirmed by a coculture of SF-GS in the anode of a microbial fuel cell (Wang et al., 2021).

Succinyl-CoA synthetase subunits (SucA; GSU2449, SucB; GSU2448, SucC; GSU1058, and SucD; GSU1059) were mostly more abundant in the SF-GS coculture (2-, 2-, 31-, and 58-fold, respectively) compared to the GS pure culture (Figure 5). In the previous studies, GS in both types of cocultures (PC-GS and GM-GS) showed low expression of succinyl-CoA synthetase genes compared to the other enzymes of the CAC (Shrestha et al., 2013). This enzyme is one of the key enzymes of the CAC that reversibly catalyzes succinyl-CoA to succinate. However, in acetate oxidation, this enzyme is not the main producer of succinate (Galushko and Schink, 2000). In previous research, succinyl-CoA synthetase was suggested to be required for carbon assimilation *via* the reversed oxidative CAC in chemolithoautotrophic growth of GS on formate or hydrogen (Zhang et al., 2020). In our earlier study, succinyl-CoA synthetase showed higher abundance in presence of hydrogen when comparing the proteome profile of GS growing on acetate, formate, and hydrogen (Mollaei et al., 2021). Therefore, the high abundance of succinyl-CoA synthetase may hint to production and consumption of hydrogen by GS, even though hydrogen was not measured in our study. Hydrogen consumption by SF-GS coculture is already shown in a propionate-fed microbial fuel cell (Wang et al., 2021). Low acetate concentrations maintained by GS in the SF-GS coculture are favorable for efficient propionate oxidation (Dong et al., 1994) but may also influence the hydrogenase and formate dehydrogenase levels required in SF, as well as the relative contribution of hydrogen and formate in electron transfer.

#### Hydrogenases and Formate Dehydrogenases of GS

**FIGURE 6:**
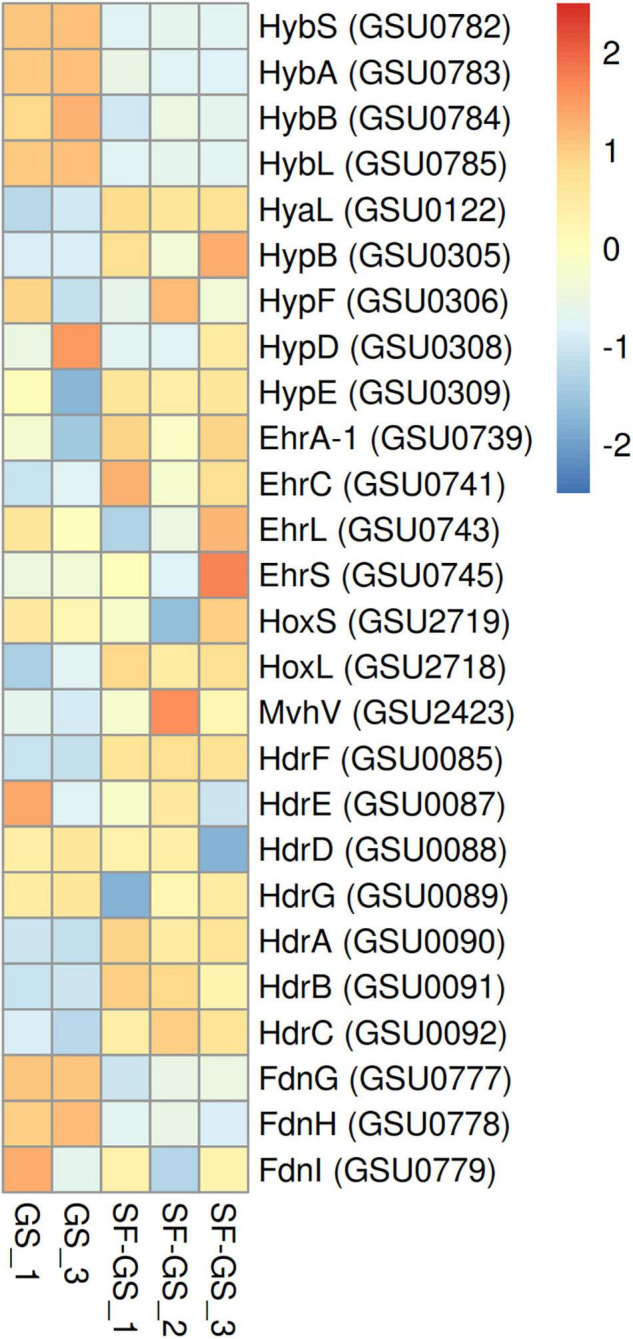
Relative abundance of the detected hydrogenases of GS in the SF-GS coculture versus the GS pure culture. Protein abundance levels are shown after *Z*-score normalization. The color intensity indicates the degree of protein presence, where high relative abundance is indicated in red and low relative abundance is indicated in blue. The rows in the heat map show the detected proteins in the SF-GS coculture and the GS pure culture. The columns show the cultures in replicates. The abbreviations of the proteins are as follows: Hyb: periplasmically oriented, membrane-bound [NiFe]-hydrogenase, small subunit (HybS), periplasmically oriented, membrane-bound [NiFe]-hydrogenase, iron-sulfur cluster-binding subunit (HybA), periplasmically oriented, membrane-bound [NiFe]-hydrogenase, cytochrome b subunit (HybB), periplasmically oriented, membrane-bound [NiFe]-hydrogenase, large subunit (HybL). Hya: periplasmically oriented, membrane-bound [NiFe]-hydrogenase, large subunit (HyaL). Hyp: hydrogenase accessory protein (HypB), hydrogenase maturation protein (HypF), hydrogenase expression/formation protein (HypD), hydrogenase expression/formation protein (HypE). Ehr: Ech-hydrogenase-related complex, NuoL-like integral membrane subunit (EhrA-1), Ech-hydrogenase-related complex, HyfE-like integral membrane subunit (EhrC), Ech-hydrogenase-related complex, large subunit (EhrL), Ech-hydrogenase-related complex, small subunit (EhrS). Hox: bidirectional NAD-reducing hydrogenase, small subunit (HoxS), bidirectional NAD-reducing hydrogenase, large subunit (HoxL). Mvh: methyl-accepting chemotaxis sensory transducer (MvhV). Hdr: heterodisulfide oxidoreductase, NAD(P)H oxidoreductase subunit F (HdrF), heterodisulfide oxidoreductase, iron-sulfur cluster-binding subunit E (HdrE), heterodisulfide oxidoreductase, iron-sulfur cluster-binding subunit D (HdrD), heterodisulfide oxidoreductase, iron-sulfur cluster-binding subunit G (HdrG), heterodisulfide oxidoreductase, FAD-binding and iron-sulfur cluster-binding subunit A (HdrA), heterodisulfide oxidoreductase subunit B (HdrB), heterodisulfide oxidoreductase, iron-sulfur cluster-binding subunit C (HdrC), Periplasmically oriented, membrane-bound formate dehydrogenase, major subunit, selenocysteine-containing (FdnG), periplasmically oriented, membrane-bound formate dehydrogenase, iron-sulfur cluster-binding subunit (FdnH), periplasmically oriented, membrane-bound formate dehydrogenase, cytochrome b subunit (FdnI). Clustering shows that samples grouped according to treatments even when only enzymes in this set are considered (Supplementary Figure 6).

GS has only one hydrogenase, a membrane-bound respiratory Hyb hydrogenase with a periplasmic-oriented active site that functions as uptake hydrogenase (Coppi et al., 2004). The abundance of all five Hyb subunits (GSU0782–0786) were significantly lower (>36-fold) in the SF-GS coculture compared to the GS pure culture growing on hydrogen, acetate, and formate (Figure 6). This is consistent with the significantly lower transcript abundance of Hyb subunit genes (>7-fold) in the GM-GS coculture compared to the PC-GS coculture (Shrestha et al., 2013).

Other hydrogenases such as Hya (GSU0120–0123), Hyp (GSU0305–0309 and GSU0374), Hox (GSU2717–2722), Mvh (GSU2416–2423), Hdr (GSU0085–0092), and Ehr (GSU0739–0745) that might be involved in hydrogen metabolism and not in hydrogen uptake (Coppi et al., 2004; Tremblay and Lovley, 2012) showed higher abundance in the SF-GS coculture compared to the GS pure culture (Figure 6). These findings suggest that hydrogen may not serve as the main shuttle compound to transfer electrons from SF to GS. Moreover, these low levels of Hyb subunits might explain a low rate of propionate conversion in the SF-GS.

To investigate if formate can function as an alternative interspecies electron carrier or together with hydrogen in the SF-GS coculture, protein abundance of all four formate dehydrogenase subunits (FdnG; GSU0777, FdnH; GSU0778, FdnI; GSU0779, and FdhD/mobA-2; GSU0780) was evaluated in this study. Interestingly, the abundance of formate dehydrogenase subunits was also significantly lower (45-, 20-, and 2-fold, respectively) in the SF-GS coculture compared to the GS pure culture, and FdhD/mobA-2 was not even detected in the SF-GS coculture (Figure 6). This further indicates that formate was unlikely an important carrier in interspecies electron transfer in the SF-GS coculture. In line with this, transcript abundance of formate dehydrogenase genes was also low in the GM-GS coculture versus the PC-GS coculture (Shrestha et al., 2013). Concluding, the significantly low abundance of uptake hydrogenase and formate dehydrogenase in electron-accepting partner suggests that both hydrogen and formate are not important electron transfer carriers or their low levels are needed in the SF-GS cocultures.

#### Electron Transport Proteins of GS

To reduce Fe(III) in the propionate-fed syntrophic coculture of SF-GS, electrons from propionate should be first transported, directly or through electron carriers (formate and hydrogen), to GS and then to Fe(III). This is likely mediated by respiratory chains consisting of *c*-type cytochromes of the inner membrane (e.g., MacA), periplasmic *c*-type cytochromes (e.g., PpcA and PpcB), outer membrane cytochromes (e.g., OmcB and OmcS), and menaquinone (MQ) that finally delivers electrons to Fe(III). Formate and hydrogen oxidation likely directly contribute to the proton motive force and electron flow to the MQ pool in the inner membrane of GS (Mollaei et al., 2021). The direct transfer of electrons between microbes has been shown to be mediated *via c*-type cytochromes located in the outer cell surface (Shrestha et al., 2013; Rotaru et al., 2014a) and *via* electrically conductive pili (Malvankar et al., 2011; Shrestha and Rotaru, 2014).

**FIGURE 7:**
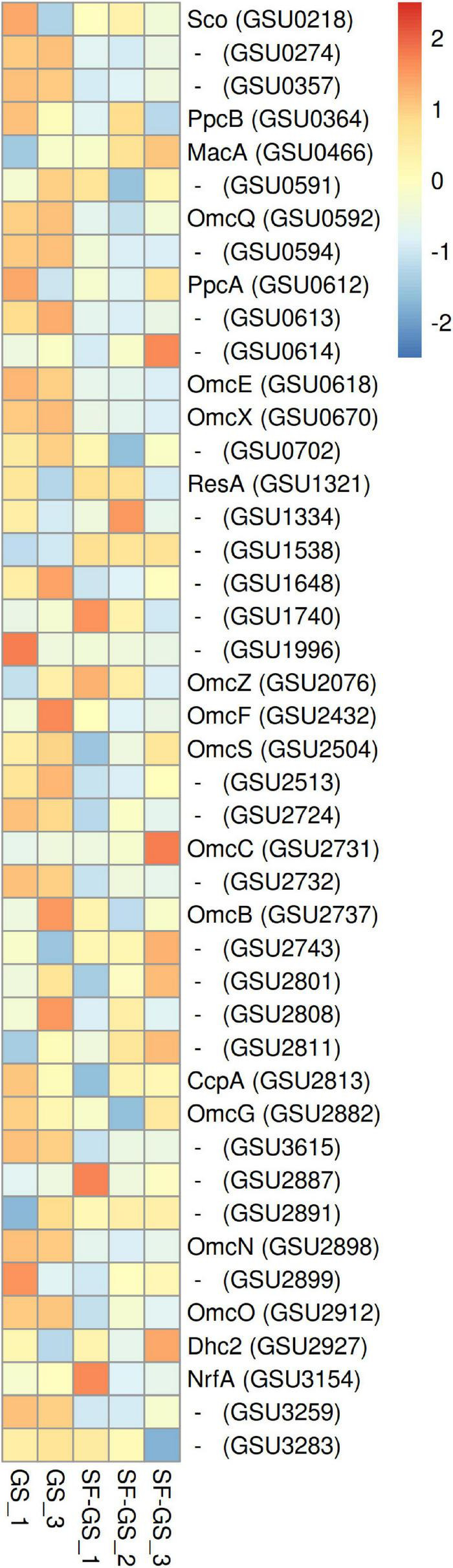
Abundance levels of the detected *C*-type cytochrome of GS in the SF-GS coculture versus the GS pure culture. Protein abundance levels are shown after *Z*-score normalization. The color intensity indicates the degree of protein presence, where high relative abundance is indicated in red and low relative abundance in blue. The rows in the heat map show the detected proteins in the SF-GS coculture and the GS pure culture. The columns show the cultures in replicates. Protein abbreviations are as follows: cytochrome c oxidase, coo3-type, synthesis factor (Sco), cytochrome c/cytochrome b (GSU0274), cytochrome c nitrite reductase (GSU0357), cytochrome c (PpcB), cytochrome c peroxidase (MacA), cytochrome c (GSU0591), lipoprotein cytochrome c (OmcQ), cytochrome c (GSU0594), ResB-like family cytochrome c biogenesis protein (GSU0613), ResC/HemX-like cytochrome c biogenesis membrane protein (GSU0614), cytochrome c (OmcE), lipoprotein cytochrome c (OmcX), lipoprotein cytochrome c (GSU0702), apocytochrome c disulfide reductase lipoprotein ResA (ResA), cytochrome c (GSU1334), cytochrome c (GSU1538), cytochrome c (GSU1648), cytochrome c, 1 heme-binding site (GSU1740), cytochrome c (GSU1996), cytochrome c (OmcS), lipoprotein cytochrome c, 1 heme-binding site (GSU2513), cytochrome c (GSU2724), lipoprotein cytochrome c (OmcC), lipoprotein cytochrome c (OmcB), cytochrome c, 1 heme-binding site (GSU2743), cytochrome c (GSU2801), lipoprotein cytochrome c (GSU2808), cytochrome c (GSU2811), cytochrome c peroxidase (CcpA), cytochrome c (OmcG), cytochrome c (GSU3615), lipoprotein cytochrome c (GSU2887), ResB-like family cytochrome c biogenesis protein (GSU2891), lipoprotein cytochrome c (OmcN), lipoprotein cytochrome c (GSU2899), cytochrome c (OmcO), cytochrome c (Dhc2), cytochrome c nitrite and sulfite reductase, catalytic subunit lipoprotein (NrfA), cytochrome c (GSU3259), and ResC/HemX-like cytochrome c biogenesis membrane protein (GSU3283). Clustering shows that samples grouped according to treatments even when only enzymes in this set are considered (Supplementary Figure 6).

MacA (GSU0466), an inner membrane *c*-type cytochrome of GS, was detected with higher abundance (7-fold) in the SF-GS coculture compared to the GS pure culture (Figure 7). This may indicate the cytoplasmic oxidation of electron donors in GS (mainly acetate due to much higher abundance of enzymes involved in the acetate oxidation than those of formate and hydrogen in the SF-GS coculture), and extracellular electron transport from the inner membrane.

To transfer electrons across the periplasm from electron donors that are metabolized in the cytoplasm, several small periplasmic cytochromes (Ppc) are involved. Of the five closely related periplasmic *c*-type cytochromes of GS (PpcA-E) (Methe et al., 2003), only PpcA (GSU0612) and PpcB (GSU0364) were detected in the SF-GS coculture (Figure 7). PpcA showed slightly higher abundance (1-fold) in the SF-GS coculture, whereas PpcB showed lower abundance (1-fold) in the SF-GS coculture as opposed to GS pure culture (Figure 7). This is in agreement with a previous study where *ppcA* showed higher transcription in PC-GS (3-fold) versus GM-GS, and *ppcB* showed lower transcription in both cocultures (Shrestha et al., 2013).

Among the outer membrane cytochromes, OmcS (GSU2504) was reported to be essential for DIET in the adapted coculture of GM-GS (Summers et al., 2010). Gene transcript abundance of OmcS in GS showed the highest increase (>308-fold) in GM-GS cocultures compared to PC-GS (Shrestha et al., 2013). This high abundance of OmcS in GM-GS cocultures was previously proposed by point mutations in the gene coding for PilR (GSU1495) during adaptation (Juárez et al., 2009; Summers et al., 2010). PilR is a transcriptional regulator for pilin and some outer membrane cytochromes, and it is required for optimal extracellular electron transfer (*via*, e.g., OmcB and OmcS) in GS (Juárez et al., 2009; Summers et al., 2010). The abundance of PilR gene transcripts was reported to be low (4-fold down) in the GM-GS coculture versus the PC-GS coculture (Shrestha et al., 2013). In another study, deletion of pilR resulted in increased expression of OmcS, but also decreased expression of the gene for PilA (GSU1496), the structural pilin protein (Juárez et al., 2009). In our study, PilR (1-fold), OmcS (2-fold), and PilA (5-fold) all showed lower abundance in the SF-GS coculture compared to the GS pure culture (Figure 7 and Supplementary Figure 6), suggesting a complex regulation mechanism for PilR.

OmcB (GSU2737), another outer membrane multiheme *c*-type cytochrome that is required for optimal Fe(III) reduction in GS, had lower abundance in the SF-GS coculture (5-fold) compared to the GS culture (Figure 7). This is consistent with lower expression of OmcB in GM-GS versus PC-GS (Shrestha et al., 2013). The expression of some outer surface cytochromes such as OmcZ (GSU2076) were downregulated (>6-fold) in a DIET-performing coculture of GM-GS compared to the PC-GS coculture (Shrestha et al., 2013). In contrast, OmcZ showed higher abundance (20-fold) in the SF-GS coculture compared to the GS pure culture (Figure 7). Some cytochromes with unknown function showed higher abundance in the SF-GS coculture than the GS pure culture (e.g., GSU2743;11, GSU2811;11, GSU1740;3, GSU2801;2, GSU2891; 2-fold higher) (Figure 7). It is not known if these proteins play a role in IET.

Based on the genetic evidence, cytochromes are more likely than pili to mediate DIET in syntrophic interactions of *Geobacter* and methanogens (Liu et al., 2018). However, the type IV pili were shown to be essential for DIET, as knocking out the gene for the structural pilin subunit PilA from GS or GM prevented coculture growth (Summers et al., 2010; Rotaru et al., 2014a,b). Both pilin A proteins (PilA-N; GSU1496, pilA-C; GSU1497) showed lower abundance (5- and 3-fold lower, respectively) in the SF-GS coculture compared to the GS pure culture (Supplementary Figure 5). Consistent with this, transcript abundance of pilin A was 5-fold lower in the GM-GS coculture than the PC-GS coculture (Shrestha et al., 2013). Among pili, some (PilM; GSU2032, PilT-1; GSU0146, PilS; GSU1494) showed higher abundance (4-, 5-, and 1-fold, respectively) in the SF-GS coculture than the GS pure culture (Supplementary Figure 5).

Overall, the knowledge about DIET is still expanding and the mere presence of DIET-associated proteins cannot guarantee occurrence of DIET (Rotaru et al., 2015; Liu et al., 2018; Otwell et al., 2018; Van Steendam et al., 2019).

## Conclusion

Here, we report a successful coculture of SF-GS on propionate and Fe(III). Neither of the two bacteria was capable of growth on propionate and Fe(III), indicating a syntrophic interaction. Using proteomic analyses, we detected low abundance of outer-surface *c*-type cytochromes and electrically conductive pili (e-pili) of GS that act as a Fe(III) reductase and as an electron carrier to other acceptors or to syntrophic partner bacteria respectively (Summers et al., 2010; Shrestha et al., 2013; Rotaru et al., 2014a,b; Ueki et al., 2018). Moreover, no aggregates were observed. Therefore, the assumption was that this syntrophic cooperation was likely based on HFIT. On the other hand, we noted significantly low abundance of hydrogenases and formate dehydrogenases in both partners. Low rate of propionate conversion (Figure 2C) coincides with the low abundance of hydrogenases and formate dehydrogenases. Furthermore, it might be that hydrogen and formate transfer is more efficient because Fe(III) is a better electron acceptor than methanogens. To clarify this, the extent of interspecies of hydrogen and formate transfer rate needs to be measured, which has not been possible so far. Therefore, further physiological and gene knockout studies are required to determine if cytochromes/pilin directly contribute to DIET. For instance, PilE/PilQ-deficient mutant of SF cocultures with GS could reveal whether it is related to IET. The results presented here and the comparison with previous studies also suggest that the proteomic profiles can reveal different patterns in physiology of cocultured members depending on the syntrophic partner. Considering the importance of GS in the bioelectrochemical field, this syntrophic interaction between SF and GS might be an alternative strategy to convert propionate into electrical current. Moreover, this collaboration helps to better understand the metabolic interactions in propionate degradation in anaerobic digestion systems.

## Data Availability Statement

The original contributions presented in the study are included in the article/Supplementary Material, further inquiries can be directed to the corresponding author.

## Author Contributions

MM, CP, and AS conceptualized and designed the experiments. MM, VS-N, and SB performed the experiments. MM, MS-D, and SB analyzed the data. All authors wrote the manuscript.

## Conflict of Interest

The authors declare that the research was conducted in the absence of any commercial or financial relationships that could be construed as a potential conflict of interest.

## Publisher’s Note

All claims expressed in this article are solely those of the authors and do not necessarily represent those of their affiliated organizations, or those of the publisher, the editors and the reviewers. Any product that may be evaluated in this article, or claim that may be made by its manufacturer, is not guaranteed or endorsed by the publisher.

## Footnotes

1
http://www.uniprot.org
