# Supplementary material for: Proteomic Analysis of a Syntrophic Coculture of Syntrophobacter fumaroxidans MPOBT and Geobacter sulfurreducens PCAT
Source: Front Microbiol. 2021 Nov 30;12:708911. doi: 10.3389/fmicb.2021.708911 (PMC8691401; doi:10.3389/fmicb.2021.708911)
Supplement: Supplementary file 1 [file Data_Sheet_1.pdf]

Supplementary Material

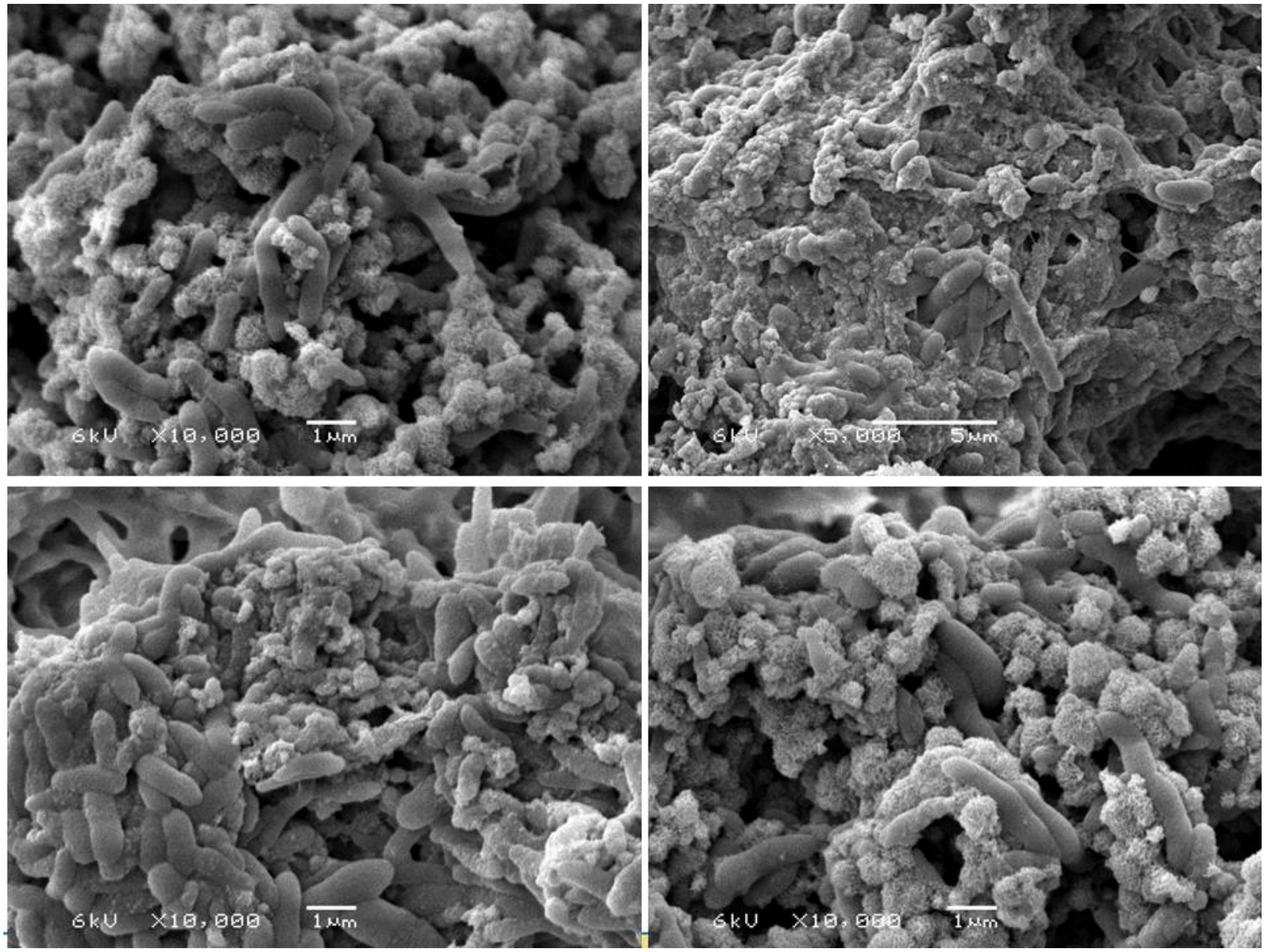

Figure S1. Scanning electron micrographs (SEM) of the SF-GS coculture.

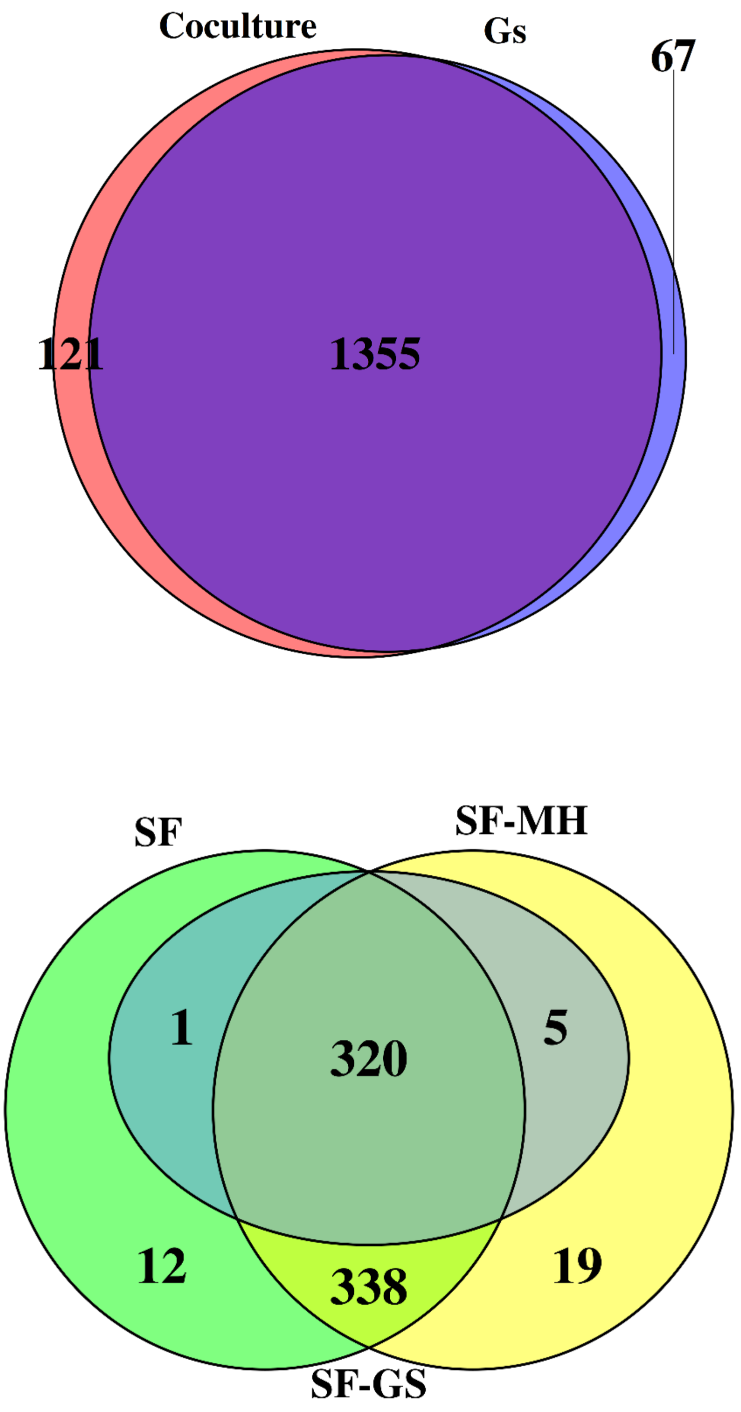

Figure S2. Venn diagram of proteins of GS detected in the SF-GS coculture and the pure culture of GS (**top panel**), and proteins of SF detected in the SF-GS cocultures, the pure culture of SF and the SF-MH cocultures (**bottom panel**). Proteins were considered when they had been detected in two samples in each experimental group.

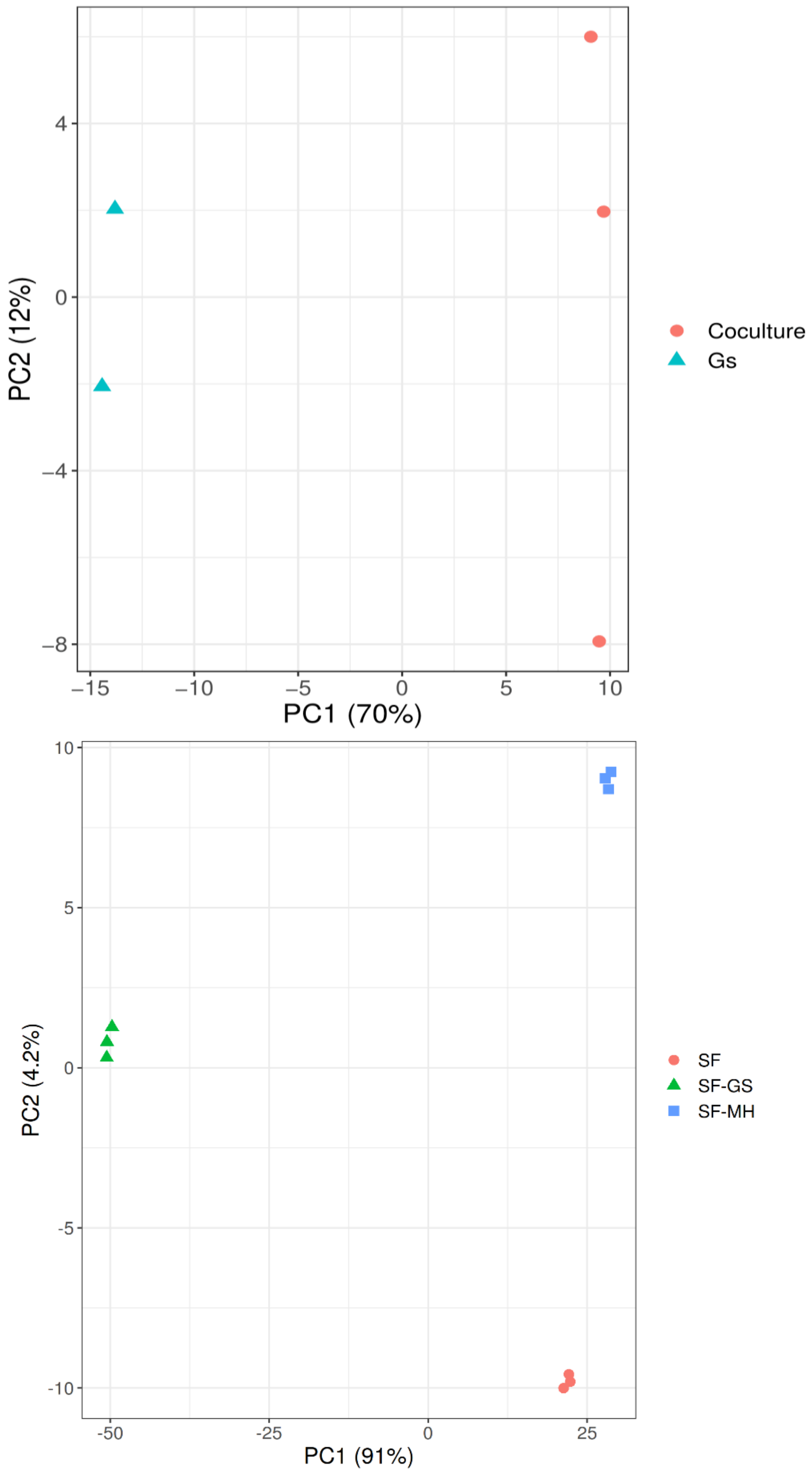

Figure S3. Principal Component Analysis (PCA) performed for the SF-GS coculture protein profiles obtained from each triplicate of the GS pure culture (**top panel**) and the SF pure culture and SF-MH cocultures (**bottom panel**).

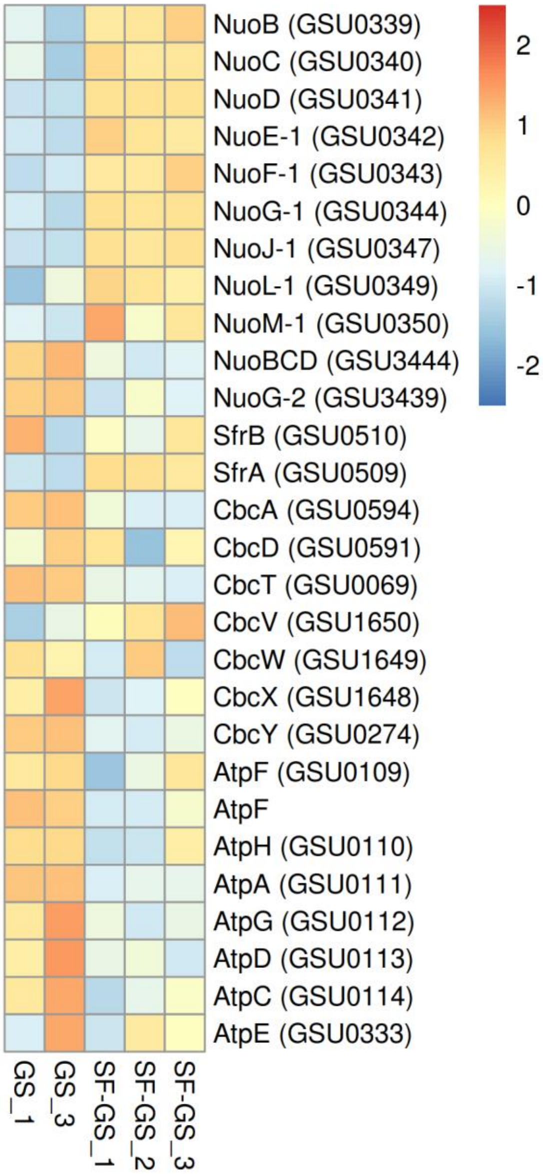

Figure S4. Relative abundance of the detected proteins of the central metabolic network of GS in the SF-GS cocultures versus the GS pure culture. Protein abundance levels are shown after Z-score normalization. The colour intensity indicates the degree of protein presence, where high relative abundance is indicated in red and low relative abundance in blue. The rows in the heat map show the detected proteins in the SF-GS coculture and the GS pure culture. The columns show the cultures in replicates. The abbreviation of the proteins are: NADH dehydrogenase I, B subunit (NuoB), NADH dehydrogenase I, C subunit (NuoC), NADH dehydrogenase I, D subunit (NuoD), NADH dehydrogenase I, E subunit (NuoE-1), NADH dehydrogenase I, F subunit (NuoF-1), NADH dehydrogenase I, G subunit (NuoG-1), NADH dehydrogenase I, H subunit (NuoH-1), NADH dehydrogenase I, I subunit (NuoI-1), NADH dehydrogenase I, J subunit (NuoJ-1), NADH dehydrogenase I, L subunit (NuoL-1), NADH dehydrogenase I, M subunit (NuoM-1), NADH dehydrogenase I, B/C/D subunits (NuoBCD), NADH dehydrogenase I, I subunit (NuoI-2), NADPH oxidoreductase, beta subunit (SfrB), NADPH oxidoreductase, alpha subunit (SfrA), Menaquinol

oxidoreductase complex Cbc5, cytochrome c subunit, putative, 7 heme-binding sites (CbcA), Menaquinol oxidoreductase complex Cbc5, cytochrome c subunit, putative, 12 heme-binding sites (CbcC), Menaquinol oxidoreductase complex Cbc5, cytochrome c subunit, putative, HAMP domain-containing, 2 heme-binding sites (CbcD), Menaquinol oxidoreductase complex Cbc4, iron-sulfur cluster-binding subunit, putative (CbcT), Menaquinol oxidoreductase complex Cbc3, iron-sulfur cluster-binding subunit, putative (CbcV), Menaquinol oxidoreductase complex Cbc3, cytochrome b subunit, putative (CbcW), Menaquinol oxidoreductase complex Cbc3, cytochrome c subunit, putative, 5 heme-binding sites (CbcX), Cytochrome c, 9 heme-binding sites, and cytochrome b (CbcY), ATP synthase F0, B' subunit (AtpX), ATP synthase F0, B subunit (AtpF), ATP synthase F1, delta subunit (AtpH), ATP synthase F1, alpha subunit (AtpA), ATP synthase F1, gamma subunit (AtpG), ATP synthase F1, beta subunit (AtpD), ATP synthase F1, epsilon subunit (AtpC), ATP synthase F0, C subunit (AtpE). The clustering was applied to verify that samples were grouped according to treatments (Fig. S7).

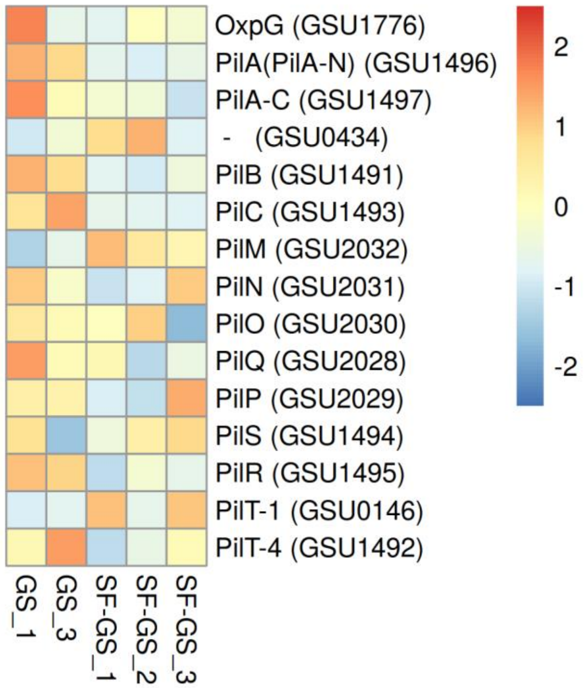

Figure S5. Relative abundance of the detected pilin proteins of GS in the SF-GS coculture versus the GS pure culture. Protein abundance levels are shown after Z-score normalization. The colour intensity indicates the degree of protein presence, where high relative abundance is indicated in red and low relative abundance in blue. The rows in the heat map show the detected proteins in the SF-GS coculture and the GS pure culture. The columns show the cultures in replicates. Protein abbreviations are: type II secretion system pseudopilin oxpG (OxpG), pilin domain 1 protein pilA (PliA-N), pilin domain 2 protein (PilA-C), thiamin biosynthesis protein ThiI-related adenine nucleotide alpha hydrolase superfamily protein (GSU0434), type IV pilus biogenesis ATPase PilB(PilB), type IV pilus inner membrane protein PilC (pilC), type IV pilus biogenesis ATPase PilM (PilM), type IV pilus biogenesis protein PilN (PilN), type IV pilus biogenesis protein PilO (PilO), type IV pilus secretin lipoprotein PilQ (PilQ), type IV pilus assembly lipoprotein PilP (PilP), sensor histidine kinase PilS (PAS, HisKA, HATPase_c) (PilS), sigma-54-dependent transcriptional response regulator PilR (REC, sigma54

interaction, HTH8) (PilR), twitching motility pilus retraction protein (pilT-1), twitching motility pilus retraction protein (PilT-4). The clustering was applied to verify that samples were grouped according to treatments (Fig. S7).

Fig. 3_tree

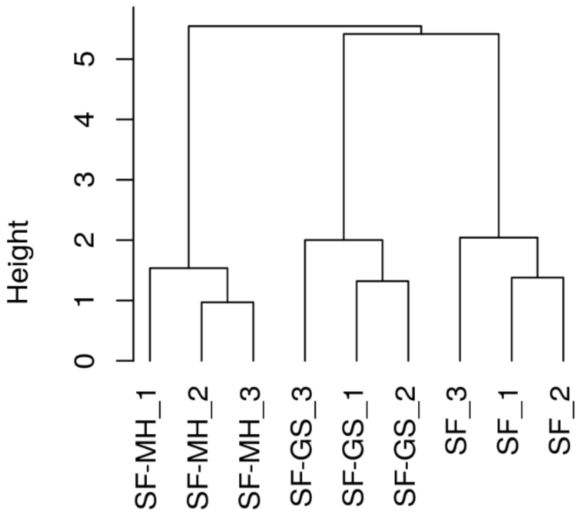

Fig. 4_tree

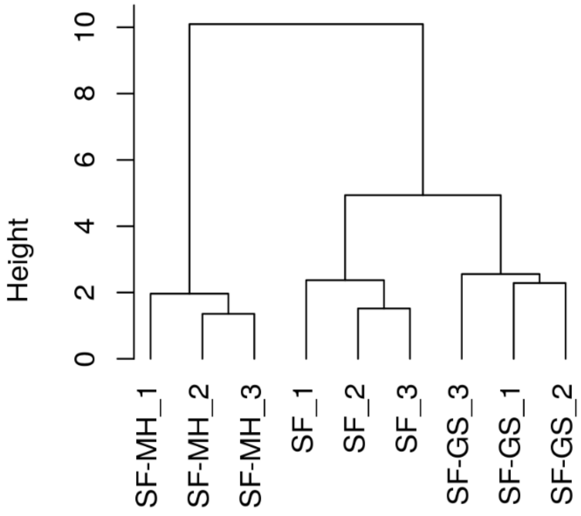

Fig. 5_tree

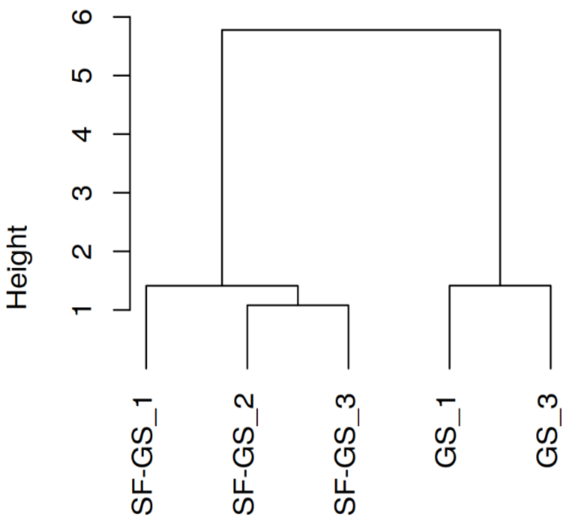

Fig. 6_tree

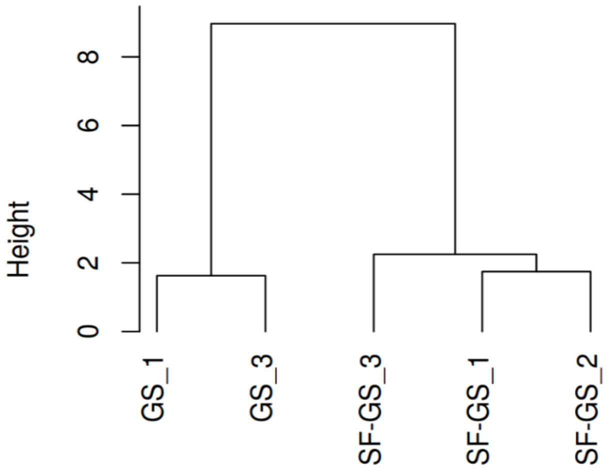

Fig. 7_tree

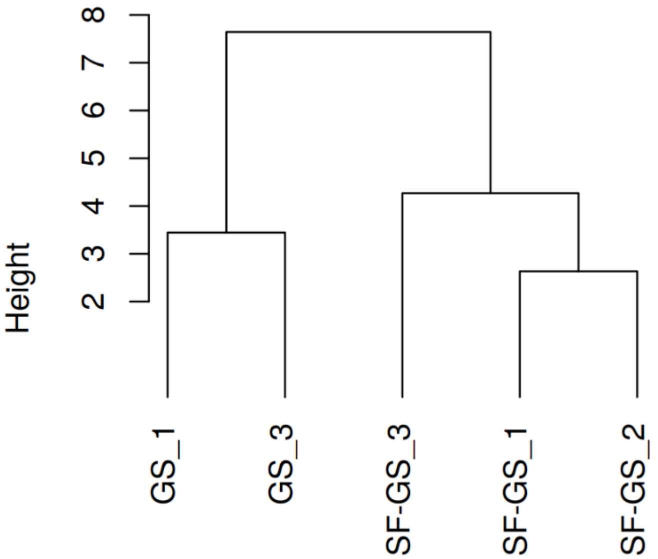

Fig. S5_tree

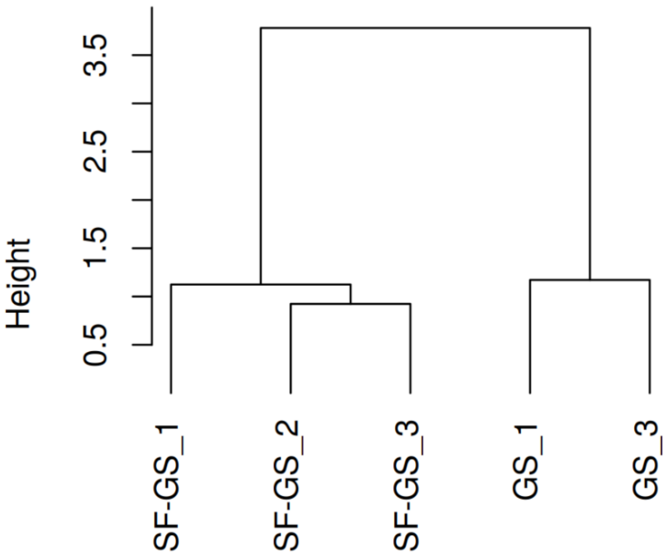

Fig. S6_tree

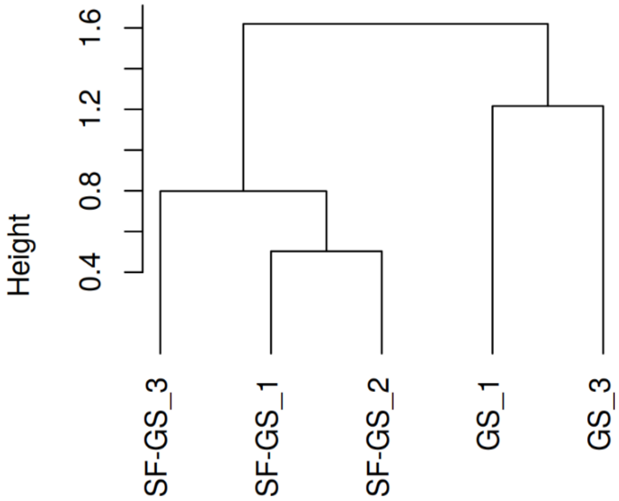

Figure S6. The dendrograms of the corresponding heatmap figures which are based on Euclidean distance between the selected samples set.
